# Early Parenting Effects on Childhood Delay of Gratification and Adolescent Allostatic Load †

**DOI:** 10.3390/children12091203

**Published:** 2025-09-09

**Authors:** Heather Leonard, Atika Khurana, Derek Kosty

**Affiliations:** 1 Department of Counseling Psychology and Human Services, University of Oregon, 1655 Alder Street, Eugene, OR 97403, USA; 2 Prevention Science Institute, University of Oregon, 1600 Millrace Dr, Eugene, OR 97403, USA

**Keywords:** adolescent allostatic load, maternal supportive presence, maternal autonomy support, maternal hostility, delay of gratification, early childhood parenting

## Abstract

**Highlights:**

**What are the main findings?**

- Parenting behaviors during early childhood (ages 2–3) can have significant, long-term associations with adolescent allostatic load.
- Both positive (supportive presence) and negative (hostility) parenting behaviors are shown to have direct associations with adolescent allostatic load.

**What is the implication of the main finding?**

- Interventions promoting supportive parenting (e.g., providing encouragement and supportive cues to the child, providing positive emotional responses to the child) and reducing hostile parenting behaviors (e.g., blaming or rejecting the child, withholding emotional support) may help improve children’s stress regulation and reduce allostatic load risk among adolescents.

**Abstract:**

**Background/Objectives**: Early parenting can influence child self-regulatory skills, which are in turn linked to allostatic load (AL)—a predictor of future morbidity and mortality. However, this mechanistic pathway from early parenting to later AL has not been tested using longitudinal data. **Methods**: We analyzed longitudinal data (*N* = 1364) from the Study of Early Child Care and Youth Development to examine relationships between early childhood (ages 2–3) observed parenting behaviors (i.e., autonomy support, supportive presence and hostility) and a comprehensive index of adolescent (age 15) AL, and assessed delay of gratification (at 54 months) as a mediator and moderator of these associations. **Results**: Maternal supportive presence and hostility in early childhood were directly associated with adolescent AL. All three early childhood parenting behaviors were associated with delay of gratification at 54 months. There was no evidence of mediation or moderation. **Conclusions**: The findings suggest the important role of early parenting behaviors in predicting long-term AL outcomes.

## 1. Introduction

Allostatic load (AL), defined as chronic wear and tear on the body due to prolonged exposure to stress, has been linked to both physical and mental health problems, including cardiovascular disease, diabetes, anxiety, and depression [1]. AL measures combine biomarkers of stress-related dysregulation across multiple biological systems (i.e., neuroendocrine, metabolic, cardiovascular, and immune systems) and are thus more reliable and better predictors of future morbidity and mortality than individual markers of stress-related dysregulation [2]. Most research on AL has been conducted in adult populations with limited focus on younger years [3]. Recent studies have shown that AL can be measured reliably in children as young as 9 years old [1], and childhood AL is predictive of long-term health outcomes, including cognitive functioning, behavioral problems, diabetes and hypertension [1,4]. Early parenting is found to play an influential role in shaping long-term health outcomes [5] by influencing the development of healthy attachment [6], self-regulation [7], and physiological systems (e.g., hypothalamic–pituitary–adrenocortical, or HPA, axis) associated with stress regulation [8]. However, long-term associations between early parenting and AL in adolescence have not been rigorously evaluated. These associations may operate directly or indirectly, through early parenting effects on child self-regulation. Parenting interventions, especially in early years, have been shown to be effective in promoting child self-regulation [7,9]. To the extent that early parenting influences adolescent AL, interventions and education designed to promote specific parenting behaviors in younger years can also have a beneficial impact on future AL and health outcomes.

There is a small body of research documenting associations between early parenting behaviors and AL markers later in development [8,10,11]. However, most of these studies have examined these associations cross-sectionally or have assessed early parenting using only retrospective reports [12]. Only a few studies have examined the associations between early parenting and adolescent AL using longitudinal samples (e.g., [8,13]), but their focus has typically been on specific biomarkers (e.g., blood pressure, awakening cortisol) and not more comprehensive AL assessments that capture dysregulation across multiple physiological systems. The present study advances current understanding of early parenting effects on adolescent AL by examining longitudinal associations between early parenting behaviors (assessed at child age 2–3 years, using observational coding of mother–child interactions) and adolescent AL, assessed at age 15 using multiple biomarkers. Further, we assessed the role of childhood self-regulation (assessed age 54 months using a delay of gratification task)—as a potential mediator and moderator of early parenting associations with adolescent AL. Given the ability of AL to predict long-term physical (e.g., cardiovascular disease, diabetes) and mental (e.g., depression, anxiety) health outcomes, it is important to study AL in adolescents and possible contributors to adolescent AL in order to promote better physical and mental health outcomes. Understanding how specific parenting behaviors in early childhood can impact adolescent AL and the underlying mechanisms of influence can enable the development of early parenting interventions that may be able to offset the risk for high AL later in development. Allostatic Load (AL).

Allostasis represents the body’s adaptive mechanism to maintain homeostasis during periods of stress [14]. AL theory, also commonly referred to as the “toxic stress” framework within the pediatric medical field [15], suggests that chronic stress exposure can lead to dysregulation of the HPA axis—the self-regulating system in the body responsible for managing how the body responds to stress [13]—contributing to downstream dysregulation of multiple body systems [16]. This primary dysregulation of the HPA axis over time leads to biological changes at the cellular level, contributing to secondary dysregulation of the inflammatory, metabolic, and cardiovascular systems, resulting in changes in key biomarkers such as blood pressure, cholesterol, and glucose levels. This, in turn, contributes to tertiary physical disease and mental health disorders [17,18].

AL is increasingly recognized as a robust predictor of future health outcomes in both children and adults [2], providing a more comprehensive assessment of stress-related dysregulation than individual biomarkers (e.g., cortisol, blood pressure). However, there is no clear consensus on which biomarkers are most relevant to include in an AL composite, especially in younger populations [14]. A recent review highlighted the wide variety of biomarkers typically utilized to calculate AL, with most studies including 6–14 biomarkers such as heart rate, blood pressure, total cholesterol, cortisol, C-reactive protein, and body mass index (BMI) [17]. In another systematic review focused on adolescent AL, the most commonly used biomarkers included cortisol, epinephrine, norepinephrine, systolic blood pressure, diastolic blood pressure, and BMI [19].

Because stress hormones are considered primary markers of dysregulation, it is recommended to include at least one primary stress hormone in the assessment of AL [19]. Cortisol, the end-product of the HPA axis, is one of the most commonly used biomarkers in AL assessments with children [1]. It is also important to include biomarkers indicating secondary changes (e.g., blood pressure, cholesterol, BMI), which could lead to tertiary disease outcomes such as cardiovascular disease [17]. A factor analysis of AL measures by King et al. [20] found that some of the best indicators to use for adolescent-specific AL include those that measure metabolic dysregulation (e.g., BMI, waist circumference, waist-to-hip ratio), suggesting that these measures may capture the earliest clinical signs of elevated AL in adolescents. Previous research with pediatric populations also emphasizes the importance of using age-specific anthropometric measures (e.g., BMI) due to the association of these measures with AL in early life [21].

There is also a lack of consensus about designating high-risk cut-off criteria for AL markers. The risk criteria cut-offs are more clearly defined for some measures (e.g., BMI) but not for others (e.g., waist-to-hip ratio). A majority of studies have used a high-risk quartile approach in which the sample distribution is divided into quartiles and a “high risk” quartile is determined, typically the highest or lowest quartile, depending on the biomarker. Some studies have used the top or bottom 10% of the distribution, representing the group at highest risk, in order to capture the most clinically significant levels for some biomarkers such as blood pressure [22,23]. When available, researchers have also used established clinical cutoffs to define high-risk [16]. For instance, BMI levels > 85th percentile for child age and sex are commonly used as the high-risk cutoff, based on current guidelines for adolescent overweight or obesity [24]. Despite the different approaches used to define high risk cutoffs in AL assessment, measurements of AL are found to be clinically reliable and valid predictors of future health outcomes [2], thus providing a screening opportunity and more targeted assessment and intervention for at-risk children and youth [25].

### 1.1. Parenting Behaviors and Adolescent AL

The childhood adversity model proposes that early childhood exposure to stressors (e.g., parental hostility, rejection, neglect) becomes biologically embedded in molecular pathways through frequent activation of stress hormones, exacerbating inflammatory responses already programmed into cells of the body, which contributes to chronic disease over time [26,27]. This biological dysregulation associated with negative parenting in early years can lead to cascading negative physical and mental health outcomes over time [28,29]. Indeed, stronger associations between AL and health problems are found among low-income children with histories of maltreatment than their peers without histories of childhood maltreatment, indicating that the effects of extreme negative parenting on AL and health outcomes are evident even in younger years [29]. Positive parenting behaviors, on the other hand, can lead to healthy development of the child’s stress response and self-regulatory systems [15]. For example, in an intervention study to promote positive parenting and maternal responsiveness, children (ages 1–3 years) of mothers who were in the intervention group had lower levels of basal cortisol one year later as compared to the control group [30]. In another longitudinal study, young adults’ (*N* = 756) retrospective recall of higher parental warmth, affection, and bonding in childhood and adolescence was significantly associated with lower AL in adulthood [31]. These findings, though primarily from adult samples, suggest that early parenting behaviors (positive and negative) may be linked to AL biomarkers across different physiological systems. Prior research has also demonstrated moderate stability in parenting behaviors from early childhood to adolescence [32,33], indicating that patterns of early parenting may be enduring with lasting impact on child health outcomes. Additional evidence also suggests that the effects of early parenting on later AL may be mediated by parenting effects on child self-regulation [8,34], however this mechanistic pathway has not been tested using longitudinal samples.

### 1.2. Parenting Behaviors and Child Self-Regulation: Mechanistic Pathways

There is a wealth of research documenting early parenting effects on the development of child self-regulation [35,36,37]. Self-regulation is broadly defined as the conscious regulation of one’s thoughts, emotions, and behaviors in the service of a goal [38]. Self-regulation develops rapidly during early childhood years, with heightened sensitivity to environmental impacts, including early parenting behaviors [7,39]. In general, positive parenting behaviors (e.g., supportive and sensitive parenting) have been linked to increases in self-regulation in early childhood years [9], while negative parenting behaviors (e.g., hostility) have been associated with self-regulation difficulties [40]. Social cognitive theories posit that supportive parenting promotes children’s self-regulation through respect for child’s autonomy, positive emotional support and responsiveness, and modeling of emotional and behavioral regulation. Over time and with practice, children become better at regulating their own emotions and behaviors. Harsh parenting, on the other hand, undermines the development of self-regulation by making it harder for the child to regulate in relational contexts characterized by heightened emotional stress, unpredictability, intrusiveness, and lack of support and sensitivity to child’s needs and autonomy [39]. Parents can thus influence children’s development of self-regulation through direct support of children’s developing regulatory capacities, modeling well-regulated behaviors, and creating a predictable, emotionally secure, nurturing environment which facilitates the development of self-regulation.

There are different dimensions of self-regulation (emotional, cognitive, behavioral) and specific parenting behaviors have been found to be associated with different measures of child self-regulation. In particular, several studies have found links between early parenting and child delay of gratification—a reliable and valid measure of behavioral self-regulation for children. Delay of gratification is typically assessed using a choice paradigm in a rewarding context, where children have to choose between an immediate reward or wait for a larger reward after a fixed amount of delay [41]. In a cross-sectional study of preschool children ages 4–6 years (*N* = 102) higher self-reported maternal warmth and support was associated with better child delay of gratification [42]. Another cross-sectional study with preschool children at age 3 (*N* = 258) found that greater maternal supportive presence (assessed using an observational task) was associated with better child delay of gratification [9]. A longitudinal study using SECCYD data found that maternal sensitivity (which included maternal supportive presence) assessed in the first three years of life is linked to greater delay of gratification at age 54 months [43]. Autonomy support, which reflects parent’s respect and support of the child’s individuality, allowing the child to actively participate in problem-solving and completing tasks with less parental intrusion, has also been positively associated with a child’s ability to delay gratification [44]. On the other hand, negative forms of parenting, such as hostility (reflecting rejection and blaming the child for mistakes), model dysregulated behaviors for the child, compromising the child’s ability to develop self-regulatory skills [45,46,47]. Previous research has found that parental hostility can create a negative coercion cycle in which harsh parenting leads to poor child self-regulation, which in turn leads to more harsh parenting, continuing to impede the development of child self-regulation over time [48].

There is evidence that both positive and negative parenting behaviors in early childhood are linked to the development of child self-regulation. To the extent that child self-regulation is linked to adolescent AL, it may be a potential mechanism by which early parenting impacts adolescent AL. As discussed previously, parenting may also influence adolescent AL biomarkers directly through biological programming that happens in younger years through early attachment in supportive and nurturing relationships [27]. For example, parenting behaviors that promote a stressful rather than supportive family environment can trigger alterations in the endocrine and autonomic nervous systems and subsequent chronic inflammation [27,34]. Early parenting effects on child self-regulation may be another mechanism through which early parenting behaviors can impact adolescent AL [49]. Children with stronger self-regulation skills, potentially linked to supportive early parenting, may be better able to handle stress effectively [2], thus leading to lower AL over time.

Individual differences in self-regulation in childhood have been linked to specific biomarkers of AL (e.g., BMI) [50], making it an important mediator to consider in the relationship between early parenting and AL. In particular, multiple longitudinal studies have found childhood delay of gratification to be linked to AL indicators such as overweight, obesity, BMI, and perceived stress [41,51]. Thus, delay of gratification may be an important mediator to examine in understanding the association between early parenting and adolescent AL. However, no study to date has examined this mechanistic pathway of influence using a longitudinal sample with objective assessments of early parenting and comprehensive AL composite.

### 1.3. Moderating Effects of Child Delay of Gratification

Individual differences in self-regulation can also operate as a moderator of contextual effects, such as early parenting, on AL outcomes [52,53]. Children with strong self-regulation skills may be able to manage stress more effectively, thus buffering the negative impacts of hostile parenting on health outcomes [54]. There is also evidence to suggest that the protective effects of positive parenting may be stronger for children with weakness in self-regulation, as they have more room to strengthen those skills and can benefit more from supportive parenting in being able to manage their emotional reactions [55]. For instance, previous research has shown moderating effects of delay of gratification in children with high negative emotionality and later AL, such that the association between high negative emotionality and AL was attenuated for those children with greater ability to delay gratification [53]. In another cross-sectional study, negative parenting was associated with a stronger cortisol response in preschool children (*N* = 160), but only for children with lower ability to delay gratification [54]. Other review studies have similarly found that children who are low in effortful control (a related dimension of self-regulation), and high in impulsivity and frustration are more susceptible to adverse consequences of negative parenting behaviors, such as the association between parental control and externalizing behaviors [56]. Given these findings related to differential susceptibility, it is possible that children who have difficulty delaying gratification may experience stronger negative impacts of hostile parenting on adolescent AL, and conversely may benefit more from positive parenting, than children with stronger delay of gratification skills. Thus, we examined whether delay of gratification operated as a moderator of the potential associations between early parenting behaviors and adolescent AL.

## 2. Materials and Methods

**Figure 1:**
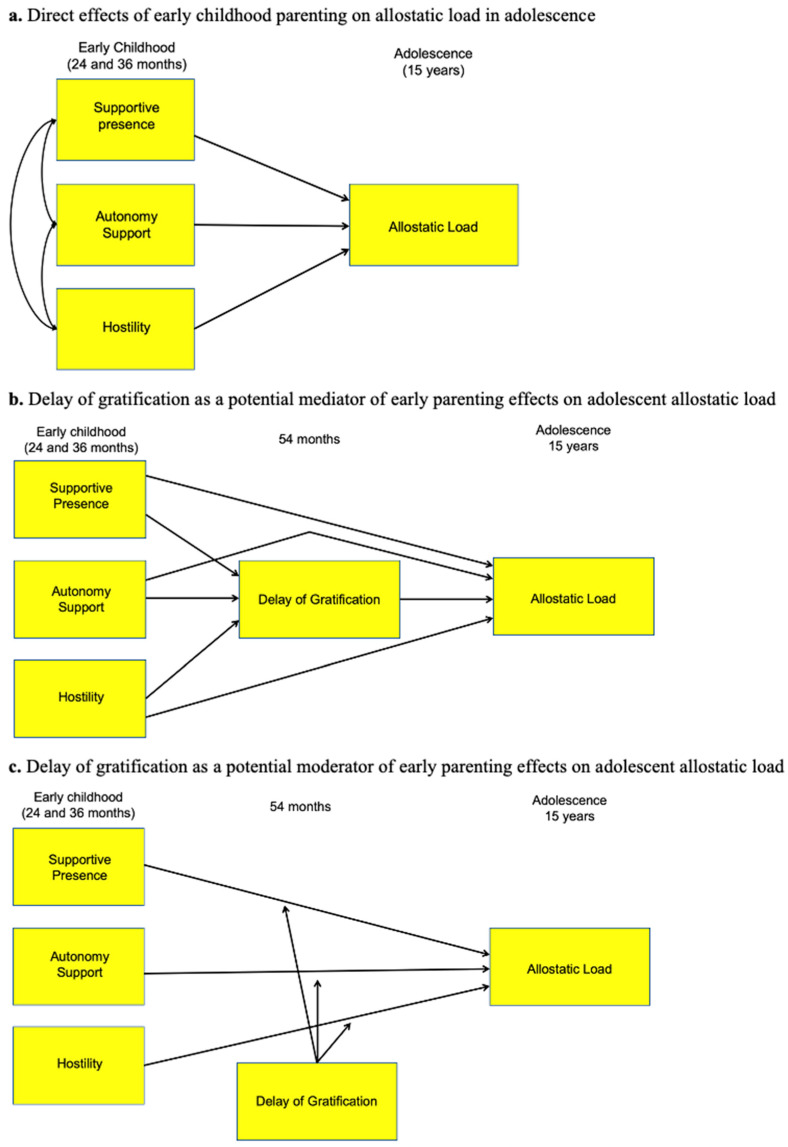
Direct and indirect effects of parenting behaviors on adolescent allostatic load. Role of delay of gratification as a potential mediator and moderator. *Note*. Direct and indirect effects of early parenting behaviors on adolescent AL will be tested together in the same model as well as separately in individual models. The moderation effects will be tested in separate models for each of the three parenting behaviors and child delay of gratification. The effects of the following covariates will be accounted for: child sex, child race/ethnicity, family income to needs ratio, maternal depressive symptoms, maternal negative life events, maternal education. These paths are omitted for clarity.

This study utilized longitudinal data from the NICHD SECCYD study to address the following research questions: (1) Is there an association between early parenting behaviors (assessed at 2–3 years of age) and adolescent AL (assessed at age 15)? (2) Does childhood delay of gratification (assessed at 54 months) mediate the associations between early parenting behaviors and adolescent AL? (3) Does childhood delay of gratification moderate the associations between early parenting behaviors and adolescent AL? We hypothesized early parenting behaviors to be significantly associated with adolescent AL, with positive parenting behaviors (i.e., supportive presence and autonomy support) being associated with lower AL and negative parenting behavior (i.e., hostility) being associated with higher AL. Further, childhood delay of gratification was expected to mediate the associations between all three parenting behaviors and adolescent AL. In particular, positive parenting behaviors were expected to be positively associated with child delay of gratification, and negative parenting (hostility) was expected to be negatively associated with child’s ability to delay gratification. Further, children with greater ability to delay gratification were expected to have lower AL scores in adolescence. Finally, childhood delay of gratification was expected to moderate the association between early parenting and adolescent AL, such that for children who exhibited greater difficulties with delaying gratification, the protective effects of positive parenting behaviors, and risk enhancing effects of negative parenting behaviors were expected to be more pronounced as compared to children who were better able to delay immediate gratification. See Figure 1 for the hypothesized model.

### 2.1. Sample Description

This study analyzed longitudinal data from the SECCYD study, which was a four-phase study conducted from 1991 to 2008 by the NICHD examining child development and childcare experiences from infancy (1 month) to mid-adolescence (age 15). The SECCYD data was uniquely suited to answer the current study questions for the following reasons: (1) being a longitudinal study spanning 15 years from birth to adolescence, it allowed us to test associations between early parenting, childhood delay of gratification, and adolescent AL, and (2) it includes objective, multi-method assessments of our key measures of interest, including observational coding of parenting behaviors at multiple time points during development, task-based assessment of child delay of gratification task at kindergarten age, when individual differences in child self-regulation can be reliably assessed [57] and have strong predictive utility [58], and a comprehensive range of biomarkers for AL assessment that were assessed by trained research staff.

Initially, 3015 eligible mothers were recruited from 24 designated hospitals across 10 U.S. cities. To be enrolled in the study, mothers had to be at least 18 years of age, English speaking, and have no reported history of substance abuse. Their infant also had to be healthy with no disability or disease at birth. The Phase I (1991–1994) sample included 1364 mothers; 65% had a high school degree or more, 11% had not completed high school, and 14% were from single-parent households (86% were from married or partnered households). Infant demographics included 52% male and 80.4% non-Hispanic White, 12.9% African American, 1.6% Asian, 0.4% American Indian/Eskimo/Aleutian, and 4.7% other.

Participating families and children completed assessments on parenting and child outcomes of interest in this study, almost annually from birth to age 15. Phase I included assessments conducted from birth to 3 years. Phase II (1995–1999) included assessments conducted from age 3 through 1st grade (child age 7 years), Phase III (2000–2004) included assessments conducted from 2nd grade through 6th grade (child age 12 years), and Phase IV (2005–2007) included assessments conducted from 7th grade through 9th grade (child age 15 years). There was 26% attrition across the waves (*N* = 1226, 1061, and 1009 across the last three phases, respectively). Attrition across the waves was higher for African American participants and those from lower SES [33]. A detailed description of the data collection procedures and instruments can be found in the study manual [59]. This study utilized data from all four phases. Assessments of early parenting behaviors were from Phase I and II; covariates were from Phase I, II, and III; childhood delay of gratification assessment was from Phase II; and adolescent AL assessments was from Phase IV of the study.

### 2.2. Measures

#### 2.2.1. Predictor Variables

**Early parenting behaviors**. Three parenting behaviors, namely supportive presence, autonomy support, and hostility, were assessed at child ages 2 and 3 years using observational coding of the semi-structured Mother–Child Interaction Task. The task was administered at multiple time points with task- and age-appropriate modifications. The interactions were recorded and then coded at a central location using 7-point rating scales. Coders were blind to all information about the dyad. Inter-rater reliability was monitored throughout the coding period with intraclass correlations ranging from 0.84 to 0.91 [33]. *Supportive presence* reflected the mother’s positive regard and emotional support for the child. *Autonomy support* was assessed based on mother’s sensitivity, respect and support for the child’s perspective and individuality [33,44]. *Hostility* scores demonstrated mother’s discounting or rejection of the child, or expression of anger towards the child.

We utilized the 24-month and 36-month assessments to capture parenting during the “early childhood” developmental stage. Earlier assessments of the mother-child interaction task (prior to the 24 month assessment) were not included because the same parenting constructs (i.e., supportive presence, autonomy support, and hostility) were not assessed during infancy. Our focus in the present study was to longitudinally examine the effects of specific parenting behaviors that have previously been linked to child self-regulation and allostatic load. In the SECCYD study, parent–child interactions assessed during infancy did not include the same parenting constructs. As such, we focused on the 2- and 3-year assessments for early parenting behaviors. For each parenting behavior, the two assessments (24 months and 36 months), coded on a scale from 1–7, were standardized and then averaged to create a continuous score. Averaged standardized scores were used to reduce measurement error associated with single time points and to capture a more stable representation of parenting behaviors during early childhood. Prior research suggests that aggregating across adjacent assessments improves reliability by smoothing short-term fluctuations and increasing construct validity [60].

#### 2.2.2. Outcome Variable

**Allostatic load (AL)**. A composite AL index was created using the following seven indicators assessed at age 15 by trained research staff: (1) systolic blood pressure, (2) diastolic blood pressure, (3) waist-to-hip ratio, (4) triceps skinfold measurement, (5) subscapular skinfold measurement, (6) BMI, and (7) awakening salivary cortisol. Measurement protocols for all variables were standardized across all study sites. Cut-off criteria for high risk levels of each variable were established based on prior research or using clinical cut-offs when available. Each variable was recoded as binary (0 = high risk criteria not met, 1 = high risk criteria met). The scores on the seven binary (0/1) variables were then added, resulting in a composite score ranging from 0–7. Due to low frequency in the higher categories, the upper category was collapsed to 5+ (i.e., scores of 5–7 were combined into one category of 5+), resulting in a range of 0–5 (*M* = 1.48, *SD* = 1.56).

**Blood pressure**. Systolic blood pressure (SBP) and diastolic blood pressure (DBP) measurements were taken at lab visits. Blood pressure was measured five times at 1-min intervals. The last three measures were averaged to create the average systolic blood pressure and average diastolic blood pressure. Values in the top decile of the sample distribution were coded as high-risk based on prior research [22,23,61].

**Waist-to-hip ratio**. Two measurements each of waist circumference and hip circumference were taken. After determining the average waist circumference and average hip circumference, waist-to-hip ratio was calculated by dividing the average waist circumference by the average hip circumference. Values in the top quartile were coded as high-risk.

**Skinfold measurements**. Three measurements of the triceps skinfold and subscapular skinfold were taken. Of the three values, if the first two measurements were identical, that value was used. If the second two measurements were identical, that value was used. Otherwise, the two closest values of the three values were averaged and used as the respective skinfold measurement (triceps and subscapular). Values in the top quartile were coded as high-risk. 

**Body mass index (BMI)**. Adolescent height and weight were measured to calculate BMI. The high risk-cut off was defined based on clinical guidelines of ≥85th percentile for child age and sex, for adolescent overweight or obesity [24]. Thirty-one percent of the sample was coded in the high-risk category (i.e., ≥85th percentile for child age and sex) for this variable.

**Awakening salivary cortisol**. After instruction on proper protocol for saliva collection, adolescents collected saliva samples upon awakening on three consecutive school days. An average cortisol value was calculated from the three daily values collected. Because lower levels of awakening cortisol are indicative of severe or enduring stress levels, values in the lowest quartile were coded as high-risk [8]. 

#### 2.2.3. Mediator and Moderator Variable

**Delay of gratification**. At 54 months, children completed a modified version of the classic marshmallow test [62]. The child was presented with a treat and told they would be alone with the treat for 7 min. The child was given the option to wait to eat the treat until the researcher returned, at which point they would receive an additional treat as a reward for waiting, or they could eat the treat before the researcher returned and not be rewarded with an additional treat. The number of seconds the child waited to eat the treat was recorded. The majority of the sample (53%) waited the full 7 min to eat the treat. Given the right censoring of distribution, the scores were re-coded to the following categories (<20 s, 20 s–2 min, 2 min–7 min, 7 min), similar to the approach used by prior studies [63] (*M* = 3.03, *SD* = 1.19).

#### 2.2.4. Covariates

**Child Sex**. Child sex has an impact on pubertal development and progression during adolescence, and child sex-based differences have been documented in case of parenting behaviors [64], and AL scores [12]. Therefore, child biological sex was included as a covariate in the model. Child sex was assessed based on mother report at the 1-month assessment (52% male, 48% female).

**Child Race–Ethnicity**. Significant variations have been observed for different racial-ethnic groups in terms of AL markers [14], therefore child race-ethnicity was included as a covariate. Due to the limited diversity, race-ethnicity was dichotomized as non-Hispanic White (80.4%) and non-White (19.6%). The non-White category included 12.9% African American, 1.6% Asian, 0.4% American Indian/Eskimo/Aleutian, and 4.7% other.

**Family income to needs ratio**. Income to needs ratio, an indicator of family socioeconomic status (SES), was included as a covariate given its associations with parenting behaviors [65] and AL [28]. Family income to needs ratio was calculated by dividing mother’s self-reported family income by the federal poverty threshold for the size of the family. The two assessments of family income to needs ratio, from child age 1 month and 36 months, were averaged to create the family income to needs ratio score used as a model covariate (*M* = 3.14, *SD* = 2.59).

**Maternal depressive symptoms**. Maternal depressive symptoms were assessed using the Center for Epidemiological Studies Depression Scale [66]. The scale included 20 depression symptoms for which mothers were asked to report the frequency with which they experienced those symptoms over the past week. Mothers completed the measure at child age 1 month, 15 months, 24 months, and 36 months. Maternal depressive symptoms in early childhood were calculated as an average of the 4 assessment time points and this average score was included as a model covariate given prior evidence of positive associations between maternal depressive symptoms and adolescent AL [67] (*M* = 10.05, *SD* = 7.02).

**Maternal negative life events**. Mothers completed the Life Experiences Survey [68] at the 54 months, grade 3, and grade 5 assessment time points. This 57-item questionnaire asked mothers to identify from a list of events (routine happenings to major and catastrophic events) those that have happened to them in the past year and the impact it had on them on a scale from +3 = very positive, 0 = neutral, to –3 = very negative. Because the data were right-skewed, scores were re-grouped into the following five categories: 1 = no life events, 2 = 1–2 life events, 3 = 3–5 life events, 4 = 6–8 life events, and 5 = 9 or more life events [69]. The three assessments at 54 months, grade 3, and grade 5 (each ranging from 1–5) were averaged to create the final negative life events score, included as a model covariate (*M* = 2.53, *SD* = 0.93). This variable was controlled for given the possible association of maternal negative life experiences and parenting behaviors.

**Maternal education**. Given that maternal education is associated with parenting behaviors, above and beyond family SES [70,71], maternal education level reported by mothers at child age 1 month was included as a covariate. Maternal education was categorized based on prior research [72] as follows: 1 ≤ 12th grade, 2 = high school graduate, 3 = some college, 4 = bachelor’s degree, 5 = graduate/professional degree) (*M* = 3.08, *SD* = 1.18).

Because these covariates have been linked to child delay of gratification and AL [43,69,73,74], the effect of the covariates was regressed on the mediator, child delay of gratification, and adolescent AL.

### 2.3. Analytic Plan

Path analysis using *Mplus* v8 [75] was used to test the main research questions. Given the count nature of the outcome variable, zero-inflated negative binomial regression was used when regressing AL on the parenting and delay of gratification variables. Patterns and distributions of missingness were evaluated to see whether the data were missing at random. Results from the Little’s MCAR Test, *X*^2^ = 82.86, df = 23, *p* = 0.000, suggested that the data were not missing completely at random (MCAR); however, multiple imputation is appropriate to use when data are not MCAR, and provides unbiased estimates [76]. Multiple imputation was used to account for missing data. Twenty imputed datasets were utilized in the analysis and the pooled results across these twenty datasets are reported. Attrition across the study period was higher for African American participants and participants with lower family income to needs ratio [33]. Both of these variables were included as covariates in the analyses. Accounting for missing data, the sample size for the regression models was 1364.

To address RQ1, the effects of all three early parenting variables were tested when included together in the same model, accounting for their covariances, and then individually in separate models to generate unadjusted results. RQ2 was evaluated by testing the effects of the three parenting behaviors on adolescent AL in separate models and including child delay of gratification as a mediator, to test for indirect effects. For RQ3, the effects of delay of gratification as a moderator were tested using joint product interaction terms for each of the parenting variables and the delay of gratification variable. Interaction terms were included in the model one at a time and tested for significance. Significance tests were examined at α < 0.05.

## 3. Results

Descriptive statistics and bivariate correlations between study variables are found in Table 1. Although all three early parenting behaviors were significantly correlated with each other (range = 0.50–0.63), multicollinearity was not an issue (VIF range = 1.04–2.57). Early maternal supportive presence and autonomy support were positively correlated (*r* = 0.56) and both supportive presence and autonomy support were negatively correlated with early maternal hostility (*r* = −0.53 and *r* = −0.63, respectively). Early maternal supportive presence and autonomy support were positively correlated with child delay of gratification at 54 months (*r* = 0.27 and 0.28, respectively), while early maternal hostility was negatively correlated with child delay of gratification at 54 months (*r* = −0.25). All three parenting behaviors were correlated with adolescent AL, with supportive presence and autonomy support being negatively correlated (*r* = −0.18 and −0.17, respectively) and hostility was positively correlated with AL (*r* = 0.17). Child delay of gratification at 54 months was also negatively correlated with adolescent AL (*r* = −0.11).

Regression output from the direct effects model, including all three early parenting behaviors in the same model accounting for their covariances, revealed no significant associations between any of the parenting behaviors and adolescent AL (see Table 2). None of the model covariates (i.e., child sex, child race-ethnicity, family income to needs ratio, maternal depressive symptoms, maternal negative life events, maternal education) had a significant effect on adolescent AL. Next, the effect of the three early parenting behaviors on adolescent AL was tested in separate models. Both supportive presence and hostility in early childhood had a significant direct effect on adolescent AL, accounting for model covariates; however, the effect sizes were small. Specifically, participants whose mothers displayed greater supportive presence in early childhood had lower levels of AL in adolescence (*B* = −0.10, SE = 0.05, ß = −0.08, *p* = 0.048), compared to those whose mothers displayed lower levels of supportive presence in early childhood (see Table 2). Further, participants whose mothers displayed high levels of hostility in early childhood were more likely to have higher AL in adolescence (*B* = 0.10, SE = 0.05, ß = 0.08, *p* = 0.04), compared to those whose mothers displayed lower levels of hostility (see Table 2). Maternal autonomy support in early childhood was not significantly associated with adolescent AL. None of the model covariates had a significant effect on adolescent AL.

Results from the mediation models revealed that the path from supportive presence (in early childhood) on child delay of gratification was significant (*B* = 0.19, SE = 0.05, *p* ≤ 0.001), but the effect from child delay of gratification to adolescent AL was not significant (*B* = −0.03, SE = 0.04, *p* = 0.34). The total indirect effect of supportive presence on adolescent AL, as mediated by child delay of gratification, was also not significant (*B*_indirect_ = −0.01, SE = 0.01, *p* = 0.37). Autonomy support (*B* = 0.20, SE = 0.06, *p* = 0.001) and hostility (*B* = −0.19, SE = 0.06, *p* = 0.001) were also significantly associated with child delay of gratification at 54 months, but the pathway from delay of gratification to AL was not significant. The total indirect effect of autonomy support (*B*_indirect_ = −0.01, SE = 0.01, *p* = 0.35) and hostility (*B*_indirect_ = −0.01, SE = 0.01, *p* = 0.40) was not significant.

For RQ3, no significant interaction effect was found between any of the three early parenting variables and child delay of gratification. The interaction effect of child delay of gratification with supportive presence (*B_int_* = −0.03, SE = 0.04, *p* = 0.48), autonomy support (*B_int_* = −0.03, SE = 0.03, *p* = 0.26), and hostility (*B_int_* = 0.03, SE = 0.03, *p* = 0.24) was not significant. None of the interaction terms were retained in the final model.

## 4. Discussion

This study examined whether parenting behaviors in early childhood are associated with AL in adolescence, either directly or indirectly through early parenting effects on childhood delay of gratification. The moderating role of childhood delay gratification was also tested. Of the three early parenting behaviors examined, supportive presence and hostility were significantly associated with adolescent AL. A unit increase in supportive presence (assessed in early childhood) was associated with a small but statistically significant decrease in AL in adolescence, and a unit increase in hostility (assessed in early childhood) was associated with a small but statistically significant increase in AL in adolescence. Although it is notable to find significant associations between early parenting and adolescent AL over a 13-year follow-up period, the magnitude of these effects was relatively small. Autonomy support was not significantly associated with AL. All three parenting behaviors (assessed at ages 2–3 years) were significantly associated with childhood delay of gratification (assessed at 54 months). However, individual differences in childhood delay of gratification did not predict adolescent AL. As such, none of the indirect effects were significant. Thus, the direct associations observed between early parenting behaviors (supportive presence, hostility) and adolescent AL were possibly due to biological embedding of early parenting effects or channeled through other potential mediators (e.g., emotion regulation). We also did not find any significant moderation effects of childhood delay of gratification suggesting that the links between early parenting and adolescent AL operated similarly regardless of children’s differences in ability to delay gratification.

Although AL is increasingly recognized as a robust marker of future morbidity and mortality, very few studies have examined associations between early parenting and AL indicators in adolescence. Further, these studies have relied on retrospective reports of early parenting or have focused on individual markers of AL instead of a comprehensive index of multiple biomarkers. The current study makes a unique contribution by examining the associations of early parenting (assessed using observational coding of mother-child interactions) with adolescent AL (assessed using a comprehensive range of biomarkers) in a longitudinal sample that was followed from birth to 15 years.

Our findings documenting direct associations between early parenting and adolescent AL while accounting for a range of sociodemographic confounds, and individual differences in child self-regulation, have important implications for prevention efforts aimed at reducing adolescent AL risk. Prior intervention studies have found that promoting sensitive parenting and positive discipline in early childhood can lower basal cortisol secretion in children at risk for externalizing behaviors [30]. To the extent that current findings are replicated in future studies with larger effect sizes, these results demonstrate a lasting physiological benefit of positive parenting behaviors (e.g., praise, physical and emotional affection, acknowledging the child’s efforts, and providing encouragement when they fail) and detriment of negative parenting behaviors (e.g., blaming, demeaning, or rejecting the child; withholding emotional support), particularly in the mother-child relationship. Intervention strategies promoting supportive parenting and reducing hostile parenting behaviors could be incorporated into parenting education as they may have utility in improving children’s stress regulation and reducing AL risk among adolescents. In addition, these findings support the American Academy of Pediatrics’ policy statement on Preventing Childhood Toxic Stress [15], demonstrating the importance of pediatricians in identifying and intervening in parent–child relationship challenges to promote positive long-term health outcomes.

Contrary to our hypothesis, autonomy supportive parenting in early childhood was not associated with adolescent AL. It is possible that some parenting behaviors matter more than others in terms of predicting future AL risk. Supportive presence is more closely aligned to the sensitive and nurturing aspects of parenting that have been linked to emotion and stress regulation, which may be a reason why it was significantly associated with adolescent AL while autonomy support was not. Parental autonomy support is more closely aligned with child executive functioning [44], and it is possible that it plays a greater role in predicting other dimensions of self-regulation like inhibitory control [77] that were not explored as potential mediators in the study. Future research should examine other potential mediators including cognitive and emotional dimensions of self-regulation and lifestyle factors (physical activity, dietary intake) to identify other potential mechanisms by which autonomy supportive parenting may be linked to future AL risk.

Exposure to harsh and negligent forms of parenting has been associated with stress dysregulation. Especially when experienced early in life, hostile parenting can lead to frequent activation of stress hormones and dysregulation of the HPA axis, as demonstrated through changes in cortisol levels [8,27,78], putting children at risk for secondary changes in biomarkers (e.g., blood pressure, cholesterol, glucose level) and tertiary changes in terms of risk for chronic disease [26,28]. Prior research using the SECCYD data has found that exposure to hostile forms of parenting between ages 4–11 was associated with greater cardiovascular risk at age 15 (e.g., higher blood pressure, BMI, and adiposity) [79]. The current study adds to this evidence base by documenting associations between hostile parenting in early childhood (ages 2–3) and AL risk in adolescence (at age 15).

Although all three early childhood parenting behaviors were significantly associated with individual differences in delay of gratification (at age 54 months), the indirect effect hypothesis was not supported because there was no significant association between childhood delay of gratification and adolescent AL. This may be related to the fact that there is only a single assessment of delay of gratification (at 54 months) in the SECCYD study, which limits our ability to assess change in this measure over time and its association with adolescent AL. It could also be that delay of gratification captures only one dimension of self-regulation (i.e., behavioral regulation in the context of a reward), and other measures (e.g., emotion regulation), or a more comprehensive assessment of self-regulation across domains (cognitive, behavioral, emotional) may operate as stronger mediators of the association between early parenting and adolescent AL. Also, it is possible that delay of gratification is more strongly related to individual AL markers such as BMI [41], and its association with comprehensive AL measures is weak. Finally, recent studies have noted limitations in the delay of gratification task in its ability to accurately assess underlying differences in self-regulation, especially in contexts characterized by risk and uncertainty [63,80]. Future research should explore other potential mediators of the link between early parenting and adolescent AL, including other measures of child self-regulation, early parent–child attachment patterns and measures of biological programming (e.g., brain functioning, HPA axis).

We also did not find any moderation effects related to individual differences in delay of gratification. Prior studies have documented interaction effects between early parenting and other dimensions of child self-regulation (e.g., effortful control) in case of externalizing behaviors [56] as well as interaction effects between early negative parenting and delay of gratification on AL-related outcomes (i.e., cortisol levels) [54]. It is possible that other aspects of self-regulation (e.g., emotion regulation, effortful control) might be better indicators of underlying differential susceptibility in effects of early parenting on adolescent AL. For example, previous research has shown child emotionality to moderate the relationship between maternal responsiveness and adolescent AL [81]. It is also possible that the moderation effects of child self-regulation are more evident in the case of specific AL markers than a comprehensive AL index. It is important to note that our models tested effects over a long developmental time-period spanning 13 years, from ages 2–3 to 15. A single time point assessment of delay of gratification at 54 months may not be enough to capture individual differences in risk susceptibility, especially when considering transactional linkages between early parenting and child self-regulation [77]. Despite these limitations, we chose the delay of gratification measure to assess individual differences in child self-regulation because (a) it is an objective, performance-based measure that is less prone to biases found in self-report measures, (b) it is a reliable and valid measure of child self-regulation in early childhood, and (c) prior studies have reported associations between childhood delay of gratification and both early parenting behaviors and adolescent AL, making it an important mediator to test. Most prior research has been cross-sectional or focused on individual AL markers (e.g., BMI). Our study extended prior findings by testing the mediating and moderating role of childhood delay of gratification in a longitudinal design and using a comprehensive measure of AL. Future studies should examine the mediating and moderating roe of child self-regulation using other measures (e.g., emotion regulation, effortful control).

The following limitations should be considered when interpreting current findings. First, it is possible that factors in parental life history (e.g., trauma) or in utero could negatively impact a child’s stress response system and AL. Information on these variables was not available to account for their effects in the current analyses. Additionally, genetic effects could not be controlled for and could account for the associations observed between parenting behaviors and child outcomes. Third, only mothers were included in this study; thus, the influence of other caregivers (e.g., fathers, grandparents) could not be examined. Fourth, the associations observed were relatively small in magnitude; however, these associations were present over a long follow-up period of approximately 13 years and were significant controlling for important covariates including family income-to-needs ratio, maternal depressive symptoms, maternal education level and negative life events. Further, parenting behaviors and adolescent AL were assessed using objective methods; as such the associations observed are less likely to be conflated due to shared method variance or reporter bias. Fifth, our measure of delay of gratification may not have adequately captured individual differences in child self-regulation and was only available at one time point. However, this measure was selected because it is a more objective assessment than teacher or mother reports of child self-regulation. Relatedly, prior research has found that high AL can lead to poor self-regulation [82], suggesting the possibility of a bidirectional relationship between self-regulation and AL. It was not possible to test these associations with the current data set as AL was only assessed at the age 15 assessment. Further, for the AL outcome variable, although seven of the most common biomarkers were utilized, we did not have an immune function indicator in the current data set (e.g., albumin, C-reactive protein, white blood cell count), which is regarded as an important indicator of stress adaptation [19]. Additionally, due to the lack of clinical high-risk cutoffs for the majority of biomarkers used in the composite AL variable, use of top/bottom quartiles could lead to quantifying some individuals as high risk even though they may not necessarily be at risk. Finally, the SECCYD sample was predominately white and socioeconomically advantaged, so the findings may not be generalizable to families from diverse racial-ethnic backgrounds or families experiencing poverty or other significant forms of adversity.

## 5. Conclusions

Overall, our findings reveal that early parenting behaviors can have significant, long-term associations with adolescent AL, observed over a 13-year follow-up period. Prior intervention studies have found that supportive and sensitive parenting behaviors can promote better stress regulation [30] and healthy behaviors (e.g., dietary intake, physical activity) in children, thereby decreasing the risk of unhealthy outcomes later in life [83]. Thus, positive early parenting behaviors may improve not only a child’s psychosocial development, but also their biology and overall physical and mental health long term. Interventions in early childhood that provide education for families to promote positive parenting behaviors such as supportive presence and decrease negative forms of parenting may help to reduce the risk of higher AL in adolescence. These findings highlight the importance of a public health approach to creating and promoting safe, stable, and nurturing parent–child relationships by supporting positive parenting education through community organizations, pediatric care, and early childhood policy [15]. We recommend that future research explore other potential mediators and moderators of the link between early parenting and adolescent AL as well as examine opportunities for further positive parenting interventions through pediatric clinics and community agencies in order to promote long-term health.

## Figures and Tables

**Table 1:** Bivariate Correlations of Study Variables.

		1	2	3	4	5	6	7	8	9	10	11
1	EC Support											
2	EC Autonomy	0.56 ***										
3	EC Hostility	−0.53 ***	−0.63 ***									
4	54 m DoG	0.27 ***	0.28 ***	−0.25 ***								
5	Adolescent AL	−0.18 ***	−0.17 ***	0.17 ***	−0.11 **							
6	Child Sex	0.08 **	0.14 ***	−0.04	0.06	−0.07						
7	Child Race/Ethnicity	−0.28 ***	−0.33 ***	0.26 ***	−0.26 ***	0.10 **	0.02					
8	Income to Needs Ratio	0.35 ***	0.27 ***	−0.25 ***	0.24 ***	−0.17 ***	0.03	−0.21 ***				
9	Maternal Depressive Sx	−0.27 ***	−0.30 ***	0.24 ***	−0.19 ***	0.12 **	−0.01	0.18 ***	−0.27 ***			
10	Maternal NLE	0.11 ***	0.11 ***	0.11 ***	−0.01	0.06	−0.02	−0.13 ***	−0.01	0.17 ***		
11	Maternal Education	0.40 ***	0.38 ***	−0.33 ***	0.27 ***	−0.18 ***	0.04	−0.22 ***	0.51 ***	−0.33 ***	0.10 ***	
	N (count)	1214	1214	1214	961	699	1364	1364	1342	1363	1154	1363
	Mean/%	−0.0018	−0.0096	0.0065	3.03	1.48	48%	19.6%	3.14	10.05	2.53	3.08
	SD	0.84	0.85	0.84	1.19	1.56			2.59	7.02	0.93	1.18

*Note*. ** *p* < 0.01, *** *p* < 0.001. EC = early childhood, Support = supportive presence, Autonomy = autonomy support, 54 m = 54 months, DoG = delay of gratification, AL = allostatic load, Sx = symptoms, NLE = negative life events. Child sex was dummy coded such that male was the omitted category. Child race-ethnicity was dummy coded such that non-Hispanic White was the omitted category. The three parental variables are standardized composite measures.

**Table 2:** Regression Estimates of Direct Effects of Early Parenting Behaviors on Adolescent Allostatic Load.

	B(SE)	β	*p* Value
Direct Effects from Combined Early Parenting Model			
Supportive Presence	−0.06 (0.06)	−0.05	0.266
Autonomy Support	0.00 (0.06)	0.00	0.997
Hostility	0.08 (0.05)	0.06	0.161
Child Sex (Female = 1)	−0.12 (0.07)	−0.06	0.079
Child Race–Ethnicity	0.059 (0.10)	0.02	0.574
Family Income to Needs Ratio	−0.04 (0.03)	−0.16	0.105
Maternal Depressive Symptoms	0.002 (0.01)	0.01	0.740
Maternal Negative Life Events	0.08 (0.04)	0.07	0.085
Maternal Education	−0.07 (0.04)	−0.08	0.087
Direct Effects: Supportive Presence Only			
Supportive Presence	**−0.10 (0.05)**	**−0.08**	**0.048**
Child Sex	−0.12 (0.07)	−0.06	0.069
Child Race–Ethnicity	0.08 (0.10)	0.03	0.466
Family Income to Needs Ratio	−0.05 (0.03)	−0.12	0.103
Maternal Depressive Symptoms	0.002 (0.01)	0.02	0.638
Maternal Negative Life Events	0.07 (0.04)	0.07	0.109
Maternal Education	−0.07 (0.04)	−0.09	0.054
Direct Effects: Autonomy Support Only			
Autonomy Support	−0.07 (0.06)	−0.06	0.219
Child Sex	−0.12 (0.07)	−0.06	0.092
Child Race–Ethnicity	0.08 (0.10)	0.03	0.470
Family Income to Needs Ratio	−0.05 (0.03)	−0.13	0.067
Maternal Depressive Symptoms	0.00 (0.01)	0.02	0.600
Maternal Negative Life Events	0.07 (0.04)	0.06	0.122
Maternal Education	−0.08 (0.04)	−0.09	0.047
Direct Effects: Hostility Only			
Hostility	**0.10 (0.05)**	**0.08**	**0.038**
Child Sex	−0.13 (0.07)	−0.07	0.051
Child Race–Ethnicity	0.07 (0.10)	0.03	0.498
Family Income to Needs Ratio	−0.05 (0.03)	−0.12	0.075
Maternal Depressive Symptoms	0.00 (0.01)	0.02	0.624
Maternal Negative Life Events	0.07 (0.04)	0.07	0.091
Maternal Education	−0.07 (0.04)	−0.09	0.060

*Note.* Values significant at *p* < 0.05 are highlighted in bold.

## Data Availability

Restrictions apply to the availability of these data. Data were obtained from the Institute for Social Research University of Michigan (ICPSR) and are available at https://www.icpsr.umich.edu/web/ICPSR/series/00233 (accessed on 2 September 2025) with permission.

## Abbreviations

The following abbreviations are used in this manuscript:

AL	Allostatic load
